# Effects of Moderate Exercise Training on Cancer-Induced Muscle Wasting

**DOI:** 10.3390/healthcare11192652

**Published:** 2023-09-29

**Authors:** Ana Cristina Corrêa Figueira, Ana Pereira, Luís Leitão, Rita Ferreira, Paula A. Oliveira, José Alberto Duarte

**Affiliations:** 1 Sciences and Technology Department, Superior School of Education of Polytechnic Institute of Setubal, 2910-761 Setúbal, Portugal; ana.fatima.pereira@ese.ips.pt (A.P.); luis.leitao@ese.ips.pt (L.L.); 2 Life Quality Research Center (CIEQV), 2400-901 Leiria, Portugal; 3 Laboratory for Green Chemistry and Technology (LAQV) of the Network of Chemistry and Technology (REQUIMTE), Department of Chemistry, University of Aveiro, Campus Universitário de Santiago, 3810-193 Aveiro, Portugal; ritaferreira@ua.pt; 4 Centre for Research and Technology of Agro-Environmental and Biological Sciences (CITAB), Department of Veterinary Sciences, University of Trás-os-Montes and Alto Douro, 5001-081 Vila Real, Portugal; pamo@utad.pt; 5 Research Center in Physical Activity, Health and Leisure (CIAFEL), Faculty of Sport, University of Porto, 4099-002 Porto, Portugal; jarduarte@fade.up.pt; 6 One Health Toxicology Research Unit (1H-TOXRUN), University Institute of Health Sciences, Campus of Gandra, 1317-116 Gandra, Portugal

**Keywords:** breast tumor, exercise training, gastrocnemius, soleus, cancer-induced muscle wasting

## Abstract

Background: Muscle wasting is a common phenomenon in oncology and seems to be attenuated by exercise training. The aim of this study is to determine the degree of aggressiveness of cancer-induced muscle wasting in two different phenotypic muscles. It will also determine whether exercise training can attenuate this muscle dysfunction. Methods: Fifty Sprague Dawley rats were randomly assigned to four experimental groups: two breast cancer model groups (sedentary and exercise) and two control groups (sedentary and exercise). Breast cancer was induced by 1-methyl-1-nitrosoureia (MNU). After 35 weeks of endurance training, animals were sacrificed, and gastrocnemius and soleus muscles harvested for morphometric analysis. Results: In sedentary tumor-bearing animals, a significant reduction in cross-sectional area was found in both muscles (*p* < 0.05). Interstitial fibrosis was significantly higher in the gastrocnemius muscle of the sedentary tumor-bearing animals (*p* < 0.05), but not in the soleus muscle. In the gastrocnemius of sedentary tumor-bearing animals, a shift from large to small fibers was observed. This cancer-related muscle dysfunction was prevented by long-term exercise training. Conclusions: In sedentary animals with tumors, the gastrocnemius muscle showed a very pronounced reduction in cross-sectional area and a marked degree of interstitial fibrosis. There was no difference in collagen deposition between tumor groups, and the soleus muscle showed a less pronounced but significant reduction in cross-sectional area. These contrasting results confirm that cancer-induced muscle wasting can affect specific types of fibers and specific muscles, namely fast glycolytic muscles, and that exercise training can be used to improve it.

## 1. Introduction

Accounting for 40–50% of total body mass in healthy, non-obese individuals, skeletal muscle is the largest organ in the human body. The function of skeletal muscle is classically defined as the ability to perform muscular contractions that generate an external mechanical force that enables the physical activities of daily living and exercise [1]. In addition, skeletal muscle plays a critical role as an essential regulator of metabolic and inflammatory homeostasis for primary and secondary disease prevention, and it is well established that the ability of exercise to prevent or slow disease progression is inextricably linked to its ability to prevent or slow muscle wasting [2]. In fact, there is substantial evidence that muscle function, defined as strength or muscle composition (muscle mass or size), is a strong independent predictor of mortality risk from all causes, cancer, and cardiovascular disease in healthy individuals [3].

In skeletal muscle, a network of signaling pathways regulates protein synthesis and protein degradation, and under certain physiological and pathological conditions, this regulation can be disrupted or disturbed, leading to skeletal muscle atrophy [4]. This atrophy results from an imbalance between protein degradation and protein synthesis and can be induced in skeletal muscle under various conditions including physical inactivity, muscle disuse, cancer, and aging [4,5]. Several lines of evidence clearly show that muscle wasting, due to reduced muscle mass and strength, is the major contributor to all-cause mortality in chronic diseases such as cancer [6,7]. Tumor-induced metabolic dysfunction, together with systemic inflammation [8], appears to affect protein turnover [9] and promotes muscle wasting, characterized by a decrease in protein content, fiber diameter, force production, and fatigue resistance, with serious implications for effective response to pharmacological treatment in cancer patients [10,11,12,13,14,15]. There is increasing evidence that loss of muscle mass is a common feature of these patients, regardless of stage of their disease [16], and that loss of appendicular lean mass is associated with increased mortality across all cancer sites [17,18,19,20].

In addition, skeletal muscle’s ability to regenerate and replace damaged myofibers with new ones, without altering the original tissue structure, depends on its ability to respond to various stimuli, such as exercise, immobilization, and disease [21]. The ability to repair itself under stress is achieved by the replacement of the original tissue with connective tissue (fibrosis), which leads to the loss of the original structure and, therefore, of its functionality [22,23,24].

In recent years, considerable efforts have been made to understand the mechanisms underlying muscle wasting and fibrosis and to find an effective agent for the treatment of both. Exercise, which downregulates the activity of pro-inflammatory cytokines and increases the phosphorylation of contractile proteins, has been proposed as a possible intervention to mitigate and/or reverse this muscle dysfunction in cancer patients [25,26]. However, there are inconsistencies in the studies that have examined the effects of cancer on muscle wasting, and it is not entirely clear which muscles are favored in the process of cancer-induced atrophy. In contrast to inactivity-induced atrophy, which mainly affects slow oxidative muscles, it is generally accepted that glycolytic muscles are more susceptible to cancer-induced muscle wasting [27,28], and selective atrophy of type II fibers, which may be accompanied by a fast to slow shift, has been reported in mouse models of cancer [29,30,31], although there are also conflicting results [32].

The present study used a mouse model of breast cancer to determine the animal’s response to endurance exercise training using two different phenotypic muscles (soleus and gastrocnemius). The aim is to investigate the role of long-term exercise training on muscle wasting, and collagen disposition, and to assess whether exercise can attenuate this and improve their regenerative capacity.

## 2. Materials and Methods

### 2.1. Animal Model and Experimental Design

The animals, 50 female Sprague Dawley mice (age 38 days; body weight 289 ± 17 g) purchased from Harlan Interfauna Inc. (Barcelona, Spain), were housed in group cages (4 animals per cage) and maintained under controlled atmospheric conditions (21–22 °C; 60% ± 5% humidity) with a 12 h dark/12 h light cycle and free access to water and food (standard laboratory chow 4RF21^®^ Mucedola, Settimo Milanese, Italy). After one week of acclimatization animals were randomly assigned to one of four experimental groups: sedentary MNU (MNU + SED, n = 15); exercise MNU (MNU + EX, n = 15); sedentary control (CONT + SED, n = 10) and exercise control (CONT + EX, n = 10). Animals in the MNU groups received an i.p. injection with 1-methyl-1-nitrosureia (ISOPAC^®^, Sigma Chemical Co., Madrid, Spain) at a dose of 50 mL/kg, while the other two groups received vehicle (saline solution 0.9%) only. Two days after carcinogen injection, animals in the exercise groups began a thirty-five-week treadmill running program (TRP) (Treadmill Control LE 8710, Harvard Apparatus, Barcelona, Spain). Duration and intensity of running were gradually increased during the first two weeks to achieve a work rate of 60 m/day, 5 days/week, at a speed of 20 m/m (an estimated intensity of 70% of maximal oxygen consumption [33]). The animal protocol was approved by the Portuguese Ethics Committee for Animal Experimentation (General Directorate of Food and Veterinary) under license number 008961. At the end of the experimental protocol, the animals were euthanized with an i.p. injection of ketamine (75 mg/kg, Imalgen^®^ 1000, Merial SAS, Lyon, France) and xylazine (10 mg/kg, Rompun^®^ 2%, Bayer HealthCare S. A., Kiel, Germany). The gastrocnemius and soleus muscles were removed, weighed, and prepared for histological analysis. Body weight was determined taking into account baseline and final body weight, and weight gain in the control groups, to characterize overall wasting [34].

### 2.2. Histology

The muscles were pinned in a paraffin plate at their in vivo length and fixed in this position for 24 h in 4% paraformaldehyde (in 0.1 M PBS, pH 7.4; containing 2.5% *w*/*v* sucrose and 0.1% glutaraldehyde). The samples were rinsed in PBS 0.1 M, pH 7.4, dehydrated in graded ethanol solutions, cleared in xylene, and paraffin-embedded. A Leica 2125 rotary microtome (Leica Microsystems Inc., Wetzlar, Germany) was used to section paraffin blocks at 5 µm. Paraffin-embedded slides were dewaxed in xylene and hydrated through a graded series of ethanol.

Light microscope: For light microscopy analysis, the slides of the gastrocnemius and soleus muscles from each animal were stained with H&E for quantitative cross-sectional area (CSA) analysis, and with PSR (Picrosirius red) for collagen content (CC) analysis.

### 2.3. Tissue Analysis

A Virtual Slide System VS110 (Olympus, Tokyo, Japan) was used to scan slides from one section of each muscle stained with PSR. Image Pro (Media Cybernetics, version 6.0) was used to analyze the digital slides to quantify collagen deposition. For CSA quantification, images were captured using a coupled digital camera (Axio Imager A1, Carl Zeiss; Jena, Germany) and analyzed using NIH ImageJ software (Image Processing and Analysis in Java; ImageJ 64-bit). An average of 1468 ± 189 fibers from each muscle and each group were analyzed for CSA quantification, and all digitized sections were used for CC.

To analyze the frequency of the fiber-size distribution, the fibers were divided into different quartiles and categorized according to their size. In the gastrocnemius muscle, the fibers measuring less than 792 μm^2^ and 977 μm^2^, for CONT + SED and CONT + EX, respectively, were categorized as small (quartile 1–Q1), and those measuring more than 1616 μm^2^/1964 μm^2^, for CONT + SED and CONT + EX, respectively, were categorized as large (quartile 3–Q3). The same procedure was used for the soleus muscle (CONT + SED–Q1 < 950 μm^2^; Q3 > 1589 μm^2^ and CONT + EX–Q1 < 1030 μm^2^; Q3 > 1751).

### 2.4. Statistical Analysis

All analyses were performed using Graph Pad Prism software (version 10.0). Kruskall–Wallis one-way ANOVA followed by Dunn’s multiple comparison test was used to test for differences between the groups’ absent normality (muscle CSA and CC and fiber diameter). Results are presented as mean ± standard deviation (SD) for weight, and as median and interquartile range (IQR) for CSA and CC. Differences were considered significant at *p* < 0.05.

## 3. Results

### 3.1. Morphometric Analysis of Gastrocnemius and Soleus Muscles

#### 3.1.1. Cross-Sectional Area

**Figure 1:**
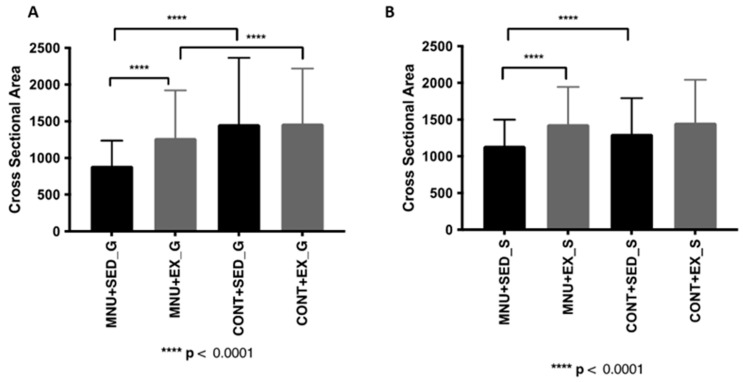
Morphometric results of the effect of exercise training on gastrocnemius. (**A**) Gastrocnemius CSA. (**B**) Morphometric results of the effect of exercise training on soleus. Abbreviations: G, gastrocnemius; S, soleus.

Although not significant, catabolic effects were observed in the gastrocnemius muscle weight of MNU animals, along with a smaller decrease in the soleus muscle (Table 1). In addition, a significant reduction in gastrocnemius cross-sectional area was observed (Figure 1A) in MNU-treated animals (*p* < 0.0001), which was greater in sedentary animals (39% vs. 13%) and was prevented by 30% by exercise training. The fiber cross-sectional area of the soleus muscle (Table 1) also shows a significant reduction but only in MNU sedentary animals (12% vs. 1%), probably related to physical inactivity rather than cancer status (Figure 1B).

#### 3.1.2. Fiber-Size Distribution

To analyze the frequency of fiber-size distribution, the fibers were divided into different quartiles and categorized by measures.

**Figure 2:**
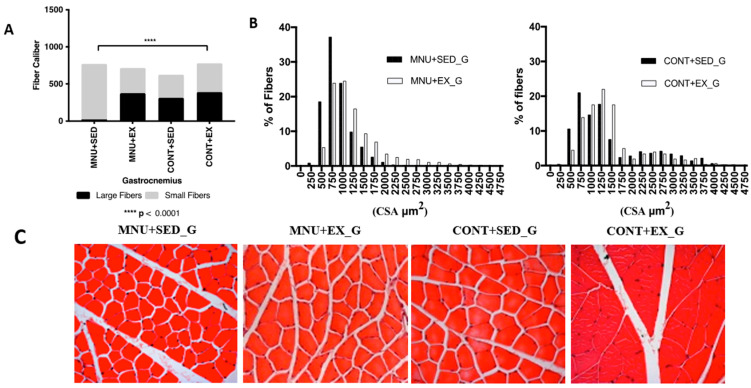
Morphometric results of the effect of exercise training on gastrocnemius. (**A**) Gastrocnemius fiber caliber. (**B**) Large and small fibers—frequency of distributions. (**C**) H&E staining from all groups (400× magnification). Abbreviations: G, gastrocnemius.

*Gastrocnemius:* The frequency distribution of the gastrocnemius fibers (Figure 2A) showed that the tumor animals suffered atrophy mainly in the large fibers. The mean fiber diameter shows a significant (*p* < 0.0001) decrease in the MNU-treated animals when compared to their control counterparts (2164 ± 244.8 μm^2^ vs. 2846 ± 577.5 μm^2^, and 2311 ± 634.5 vs. 2547 ± 677.6 μm^2^ for sedentary and exercising animals, respectively). In addition, sedentary animals with tumors had 96% fewer large fibers (1% vs. 25%) than controls. Fibers sized ≥1750 μm^2^ were almost absent and completely disappeared around 2000 μm^2^ (Figure 2B).

The effect of exercise training was observed in the higher expression of large fibers in MNU-exercised animals (Figure 2C), with 16% less than in CONT + EX (21% vs. 25%) but with 95% more than in the MNU + SED ones (21% vs. 1%).

There appears to be a shift towards smaller fibers in the gastrocnemius muscle of the MNU animals, which was partially reduced by exercise. The MNU + SED animals have more than 44% smaller fibers than the controls (45% vs. 25%), while the MNU + EX animals show a difference of 11%. We believe that this may explain the differences observed in the gastrocnemius cross-sectional area of the MNU + SED animals.

*Soleus:* The frequency of fiber-size distributions in the slow soleus (Figure 1B) also shows a significant (*p* < 0.0001) atrophy of large fibers in MNU + SED animals, although it is less pronounced (42%) than in the gastrocnemius.

**Figure 3:**
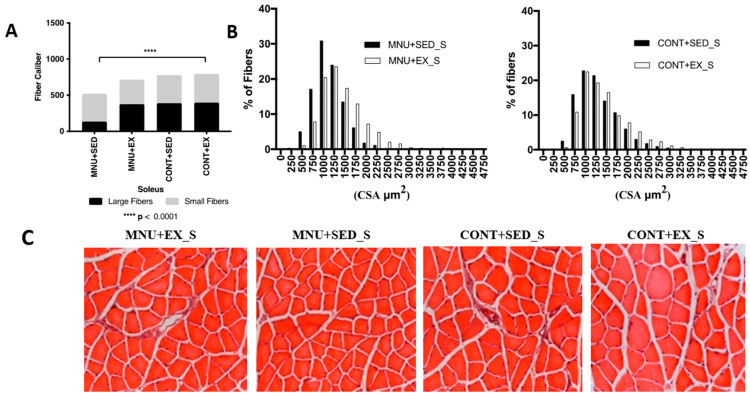
Morphometric results of the effect of exercise training on soleus. (**A**) Soleus fiber caliber. (**B**) Large and small fibers—frequency of distributions. (**C**) H&E staining from all groups (400× magnification). Abbreviations: S, soleus.

A lower expression of large fibers was observed in MNU sedentary animals (Figure 3A) when compared to control (11% vs. 25%), and a small difference (8%), probably induced by exercise, was observed between active animals (23% vs. 25%).

A shift towards smaller fibers also seems to occur in sedentary animals with tumors, although it is much less pronounced than in the gastrocnemius (Figure 3B), with 19% more expression of small fibers in MNU + SED animals compared to CONT + SED (31% vs. 25%) (Figure 3C).

The opposite results were observed between trained animals, where CONT + EX animals had 20% more small fibers than the MNU + EX ones (25% vs. 20%), which can be another argument in favor of the inactivity-induced atrophy of the slow soleus.

#### 3.1.3. Muscle Collagen Content

**Figure 4:**
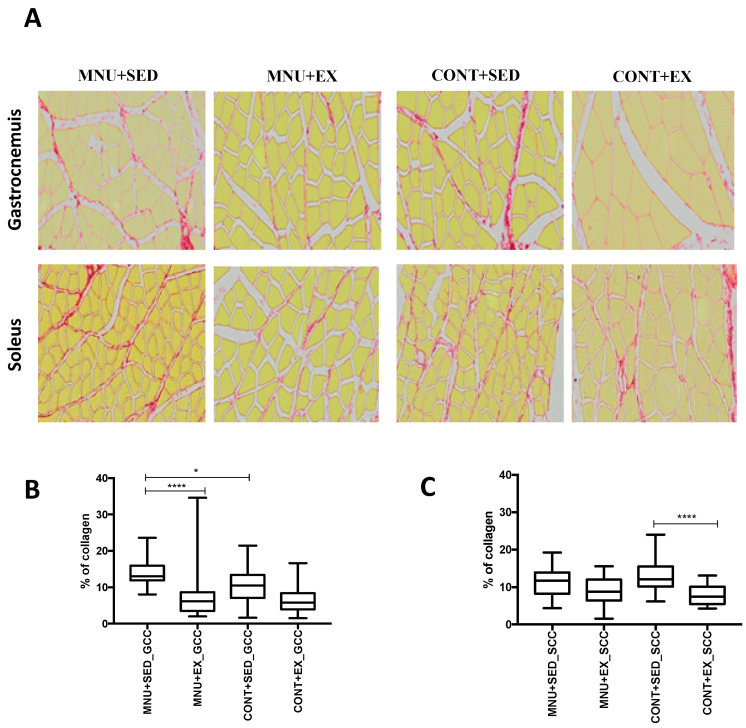
(**A**) Gastrocnemius and soleus muscle—PSR staining from all groups (400× magnification). (**B**) Data distribution of CC for gastrocnemius muscle. (**C**) Data distribution of CC for soleus muscle. * MNU+SED_GCC vs. CONT+SED_GCC (*p* < 0.05); **** MNU+EX_GCC vs. MNU+SED_GCC; CONT+SED_SCC vs. CONT+EX_SCC *p* < 0.0001). Abbreviations: G, gastrocnemius; S, soleus.

To verify the effect of exercise training on the regeneration versus fibrosis process, an analysis of collagen content was performed. The replacement of functional tissue with dense connective tissue occurred with a higher expression in the gastrocnemius than in the soleus, and exercise training seems to increase their regenerative capacity (Figure 4A).

The results showed a higher aggressiveness of tumorigenesis in the interstitial fibrosis of the gastrocnemius than in the soleus, expressed by a significant (*p* = 0.0006) increase in collagen content (45%) in MNU + SED animals (Figure 4B) when compared to trained animals (26% vs. 14%) and to controls (26%). No significant differences (*p* = 0.0980) were observed in the soleus muscle collagen deposition of the MNU groups (Figure 4C).

## 4. Discussion

Endurance exercise is a commonly performed form of exercise that significantly increases skeletal muscle energy and oxygen requirements, thereby altering metabolic regulation throughout the body. In fact, it is a well-established intervention to preserve muscle function and prevent changes in energy metabolism [35].

Using a mouse model of endurance exercise involving 35 weeks of daily treadmill running, the present results show that even without significant differences in body weight (Table 1) and without altering food consumption, the response to the cancer stimulus significantly affected the capacity and function of the muscles analyzed.

These results show that even without significant differences in body weight (Table 1), the response to the cancer stimulus significantly affected the capacity and function of the muscles analyzed. The present results also show that although no significant evidence of muscle weight was detected, there is evidence of muscle wasting in MNU animals, which could be prevented by exercise training. Given that the interest in muscle function in oncology is mainly related to the study of cachexia (a multifactorial syndrome that can be responsible for a high number of deaths in cancer patients) associated with cancer, and that a loss of more than 5–10% of body weight is generally considered to be the starting point for a cachectic state, these results confirm some of the evidence found in this field, such as the physiological changes, may be present before this threshold is reached [36]. Furthermore, it appears that the use of body weight as a measure to determine cachexia appears to be arbitrary, and diagnostic criteria should go beyond weight loss to include direct measures of body composition, particularly by monitoring muscle loss [19,37].

The animals’ response to tumor burden was different between the muscles studied, showing greater aggressiveness in the gastrocnemius, which is consistent with the results of previous studies showing that muscle atrophy in a cancerous state appears to be a phenotype-dependent phenomenon. Furthermore, it appears that muscle wasting is highly selective in targeting key muscle gene products, rather than being a process regulated by the downregulation of a general set of myofibrillar proteins [27,28,38,39].

Cross-sectional area analysis, a highly predictive parameter of muscle strength and functionality, revealed significantly increased atrophy in both muscles in MNU sedentary animals compared to all other groups, and collagen accumulation was significantly higher in the gastrocnemius but not in the soleus. Sedentary animals with tumors had a total fiber CSA that was 30% lower in the gastrocnemius and 10% lower in the soleus than in the respective controls. In the gastrocnemius of sedentary control animals, atrophy is concentrated in smaller fibers, suggesting that disuse atrophy is greater in this fiber size. In contrast, in cancer-sedentary animals, the focus is on large fibers with overexpression in the smaller ones. Although we have no data to determine whether there was a shift in fiber profile, given that the mechanisms regulating exercise training adaptations may be fiber-type dependent [40], and that the gastrocnemius is predominantly composed of fast-type fibers [18], it is possible to speculate that significant atrophy of gastrocnemius fast-type fibers would be found, accompanied by a fast to slow shift, as similar changes can be found in muscle biopsies from human cancer patients and mouse models of cancer cachexia [28,30,41,42].

Regarding the soleus, the lack of differences observed between the exercised animals (MNU + EX and CONT + EX) in the frequency of large fibers, and the lowest expression in the small fibers of the same animals, could indicate that the higher differences found in the large fibers of the sedentary animals may not reveal a cancer-induced muscle wasting, but rather a disuse-induced muscle wasting. This seems to be confirmed by the marked interstitial fibrosis found in the soleus between control groups and the lack of differences between the MNU animals. Indeed, the replacement of muscle fiber by collagenous tissue could be promoted by inactivity, resulting in the inefficiency of the regenerative and contractile capacity of skeletal muscle [21,22]. In addition, the overexpression of collagen tissue found in the extracellular matrix of the gastrocnemius muscle could be related to the reduced expression of dystrophin [43,44], which has been reported to be a type II fiber-specific condition in tumor-bearing animals [29].

In the context of skeletal muscle, adaptation is often discussed as a change in structure and function in response to exercise. The nature of the stimulus is entirely dependent on the type of adaptation that occurs in skeletal muscle in response to exercise, and much of the adaptive variation is fiber-type specific and strongly dependent on the diverse structural, metabolic, and functional characteristics of each fiber type [45]. In addition, it is now thought that skeletal muscle stem cells, located under the basal lamina of muscle fibers and also known as satellite cells, play an important role in the adaptive process. Satellite cells are altered in their proliferation and differentiation activity in cancer status and are also known to play an important role in muscle regeneration [46]. Exercise training, which is one of the most effective stimuli to induce satellite cell activation, may be another reason to explain the reduction in fibrosis in exercised animals with tumors [47]. These contrasting data on fibrosis content between the analyzed muscles are probably a further reinforcement in favor of a selective atrophic state of type II fibers in response to tumor burden.

Another possible explanation for the susceptibility of different fiber types to atrophy may be related to differences in their oxidative capacity. Skeletal muscles with different fiber types have different contractile and metabolic properties, and oxidative muscles, such as the soleus, are rich in mitochondria and capillaries, whereas glycolytic muscles, such as the gastrocnemius, are poor in these and appear to be more prone to atrophy in chronic disease conditions [48]. Oxidative fibers appear to be more resistant to cancer-induced atrophy than glycolytic fibers, probably because this type of stimulus induces less oxidative stress in the former [49]. Interestingly, this resistance to oxidative stress appears to be greater in cancer-induced atrophic fibers than in disuse fibers, suggesting that the activation of different protein degradation/synthesis pathways may depend on the type of stimulus [11,39]. In the present study, we were able to confirm that the gastrocnemius is more susceptible to the cancer-induced muscle wasting than the soleus, and we also confirmed that the endurance exercise training performed, had a positive effect on muscle wasting, as indicated by the differences in the cross-sectional area of the trained animals, along with their regenerative capacity.

Our results also showed that despite the lack of cachectic evidence, as shown by the small differences in the weight found in tumor-free animals compared to tumor-bearing animals, skeletal muscle wasting can occur in cancer and that exercise training may act as a tool to prevent it, which is consistent with the results of several studies suggesting early intervention in the treatment of muscle wasting with a combination of resistance and endurance training [50,51] to improve fatigability, reduce inflammation, and increase muscle protein synthesis [52].

## 5. Conclusions

Skeletal muscle is the most abundant tissue in healthy individuals and its importance extends beyond voluntary movement, and any weakness in skeletal muscle results in some degree of instability and inefficiency. Among other things, disease is one of the factors that can contribute to the impairment of skeletal muscle function, which can adversely impact the quality of life. Given their important role in health and quality of life, it is crucial to understand the conditions under which muscles respond positively to the atrophic stimulus. Our results show that endurance training is an important intervention to reverse cancer-related muscle wasting. In fact, since the active animals with tumors showed significantly less muscle wasting than the sedentary animals that also had tumors, these results show that tumor status appears to induce greater muscle wasting than disuse, and that endurance exercise appears to reverse this. In addition, it is possible to confirm the different degrees of aggressiveness induced by this cancer status in these fast and slow muscles, and that the larger fibers are the chosen target. Together, our results provide a deeper understanding of the cancer-associated muscle-wasting phenotype in a mouse model, which may be useful in the fight against muscle wasting in this disease. The ability to regenerate muscle tissue was also improved in trained animals, showing that exercise is a powerful tool for muscle regeneration and growth even in stressful conditions.

We believe that this study will provide new insights into the process of muscle wasting in breast cancer. Identifying the factors responsible for the differential response of muscle fiber types may lead to a better understanding of the different conditions that lead to muscle wasting and thus help in the design of therapeutic interventions appropriate for specific disorders.

In conclusion, these data show that by modulating a shift in fiber size and reducing fibrosis, 35 weeks of endurance training can ameliorate cancer-induced gastrocnemius muscle wasting in an animal model of breast cancer.

Future studies should focus on understanding whether different types of training, namely resistance training, which targets a specific type of muscle and a specific type of fibers, can prevent the wasting of large fibers in glycolytic muscles.

## Figures and Tables

**Table 1:** Response of the animals to the exercise training. Body weight and skeletal muscle weight expressed as mean ± standard deviation. Cross sectional area (CSA) of muscle fibers and collagen content (CC), from gastrocnemius and soleus muscles, expressed as median with [interquartile range]). Results from MNU (N-methyl-N-nitrosourea) groups and control groups. * vs. MNU + EX; ^¥^ vs. CONT + SED; ** vs. CONT + EX (*p* < 0.05).

	MNU Groups		Control Groups	
	Sedentary	Exercised	Sedentary	Exercised
Body weight				
Initial (g)	187.7 ± 14.3	179.5 ± 11.7	159.4 ± 7.2	154.3 ± 9.83
At sacrifice (g)	287.1 ± 13.4	294.3 ± 25.0	298.3 ± 14.4	298.2 ± 24.3
Acurate (g)	272.5 ± 13.9	281.8 ± 31.6	-	-
Gain (g)	84.8 ± 13.9	102.3 ± 31.6	138.9 ± 14.4	171.7 ± 48.7
Gastrocnemius weight (g)	3.62 ± 0.3	3.70 ± 0.6	3.92 ± 0.22	4.16 ± 0.13
Gastrocnemius CSA (μm^2^)	831–[658–1042] *^¥^	1084–[841–1472] **	1178–[792–1964]	1284–[977–1616]
Gastrocnemius CC (%)	13.01–[11.9–15.9] *^¥^	8.1–[5.4–9.25]	10.47–[7.1–13.4]	5.78–[3.9–8.4]
Soleus weight (g)	0.19 ± 0.03	0.20 ± 0.02	0.20 ± 0.02	0.23 ± 0.02
Soleus CSA (μm^2^)	1104–[903–1350] *^¥^	1347–[1078–1703]	1220–[950–1589]	1320–[1030–1751]
Soleus CC (%)	11.77–[8.3–13.9]	8.77–[6.4–12.0]	12.09–[10.2–15.5] **	7.47–[5.4–10.1]

## Data Availability

The data presented in this study are available upon request from the corresponding author.
